# Guanylate-binding protein 1 expression from embryonal endothelial progenitor cells reduces blood vessel density and cellular apoptosis in an axially vascularised tissue-engineered construct

**DOI:** 10.1186/1472-6750-12-94

**Published:** 2012-12-06

**Authors:** Oliver Bleiziffer, Matthias Hammon, Andreas Arkudas, Christian D Taeger, Justus P Beier, Kerstin Amann, Elisabeth Naschberger, Michael Stürzl, Raymund E Horch, Ulrich Kneser

**Affiliations:** 1 Department of Plastic and Hand Surgery, University of Erlangen-Nuremberg, Krankenhausstr 12 91054, Erlangen, Germany; 2 Department of Nephropathology, Erlangen University Medical Center, Krankenhausstr 12 91054, Erlangen, Germany; 3 Division of Molecular and Experimental Surgery, Erlangen University Medical Center, Krankenhausstr 12 91054, Erlangen, Germany

**Keywords:** Angiogenesis, Endothelial progenitor cells, Guanylate-binding protein 1, *In vivo* tissue engineering

## Abstract

**Background:**

Guanylate binding protein-1 (GBP-1) is a large GTPase which is actively secreted by endothelial cells. It is a marker and intracellular inhibitor of endothelial cell proliferation, migration, and invasion. We previously demonstrated that stable expression of GBP-1 in murine endothelial progenitor cells (EPC) induces their premature differentiation and decreases their migration capacity *in vitro* and *in vivo*. The goal of the present study was to assess the antiangiogenic capacity of EPC expressing GBP-1 (GBP-1-EPC) and their impact on blood vessel formation in an axially vascularized 3-D bioartificial construct *in vivo*.

**Results:**

Functional *in vitro* testing demonstrated a significant increase in VEGF secretion by GBP-1-EPC after induction of cell differentiation. Undifferentiated GBP-1-EPC, however, did not secrete increased levels of VEGF compared to undifferentiated control EPC expressing an empty vector (EV-EPC). In our *In vivo* experiments*,* we generated axially vascularized tissue-engineered 3-D constructs. The new vascular network arises from an arterio-venous loop (AVL) embedded in a fibrin matrix inside a separation chamber. Total surface area of the construct as calculated from cross sections was larger after transplantation of GBP-1-EPC compared to control EV-EPC. This indicated reduced formation of fibrovascular tissue and less resorption of fibrin matrix compared to constructs containing EV-EPC. Most notably, the ratio of blood vessel surface area over total construct surface area in construct cross sections was significantly reduced in the presence of GBP-1-EPC. This indicates a significant reduction of blood vessel density and thereby inhibition of blood vessel formation from the AVL constructs caused by GBP-1. In addition, GBP-1 expressed from EPC significantly reduced cell apoptosis compared to GBP-1-negative controls.

**Conclusion:**

Transgenic EPC expressing the proinflammatory antiangiogenic GTPase GBP-1 can reduce blood vessel density and inhibit apoptosis in a developing bioartificial vascular network and may become a new powerful tool to manipulate angiogenetic processes in tissue engineering and other pathological conditions such as tumour angiogenesis.

## Background

The role of endothelial progenitor cells (EPC) in the formation of new blood vessels has been the focus of intense research and debate over the past years but the involved mechanisms remain unclear. In pathologic conditions involving tissue ischemia such as myocardial infarction, limb ischemia, and tumor angiogenesis [1,2], EPC are chemotactically recruited from bone marrow and the peripheral blood to the site of injury where they differentiate into mature endothelial cells. These cells may contribute directly to *de novo* microvascular blood vessel formation, a process termed vasculogenesis [1]. Alternatively, they may act through a more paracrine manner by secretion of proangiogenic growth factors such as VEGF rather than integrate directly into newly formed blood vessels, [3].

At the same time, EPC recruitment and proangiogenic potential may be adversely influenced by an unfavourable microenvironment [4]. It has been previously shown that chronic inflammation and increased levels of proinflammatory cytokines induce the secretion of the large GTPase Guanylate binding Protein-1 (GBP-1) from endothelial cells. GBP-1 mediates the potent antiangiogenic effects of the cytokines TNF-α, IFN-γ and IL-1 *in vitro* and *in vivo*[5-8].

To investigate the function of GBP-1 in EPC, we previously established EPC stably expressing GBP-1 (GBP-1-EPC) and performed extensive functional *in vitro* characterization [9]. Premature differentiation as indicated by upregulation of vWF and VEGFR-2 was observed in these transgenic cells compared to control cells carrying an empty vector only. Furthermore, GBP-1 inhibited the proliferation and migration of EPC *in vitro*. EPC were then suspended in a fibrin matrix which was placed in a separation chamber containing a microsurgically created AV vessel loop. The AV loop provides a well established and highly standardized *in vivo* model of predictable blood vessel formation generated from an axial vascular axis [10,11]. After even distribution of EPC in the fibrin matrix, expression of GBP-1 prevented cell migration towards the central vessels of the construct compared to control cells [9].

Based on these findings, the aim of the current study was to perform further functional testing related to angiogenic capacity of GBP-1-EPC *in vitro* and to investigate the impact of the anti-angiogenic cytokine GBP-1 on the morphometry of the tissue-engineered construct as well as its blood vessels *in vivo*.

## Results

### VEGF secretion in vitro

**Figure 1:**
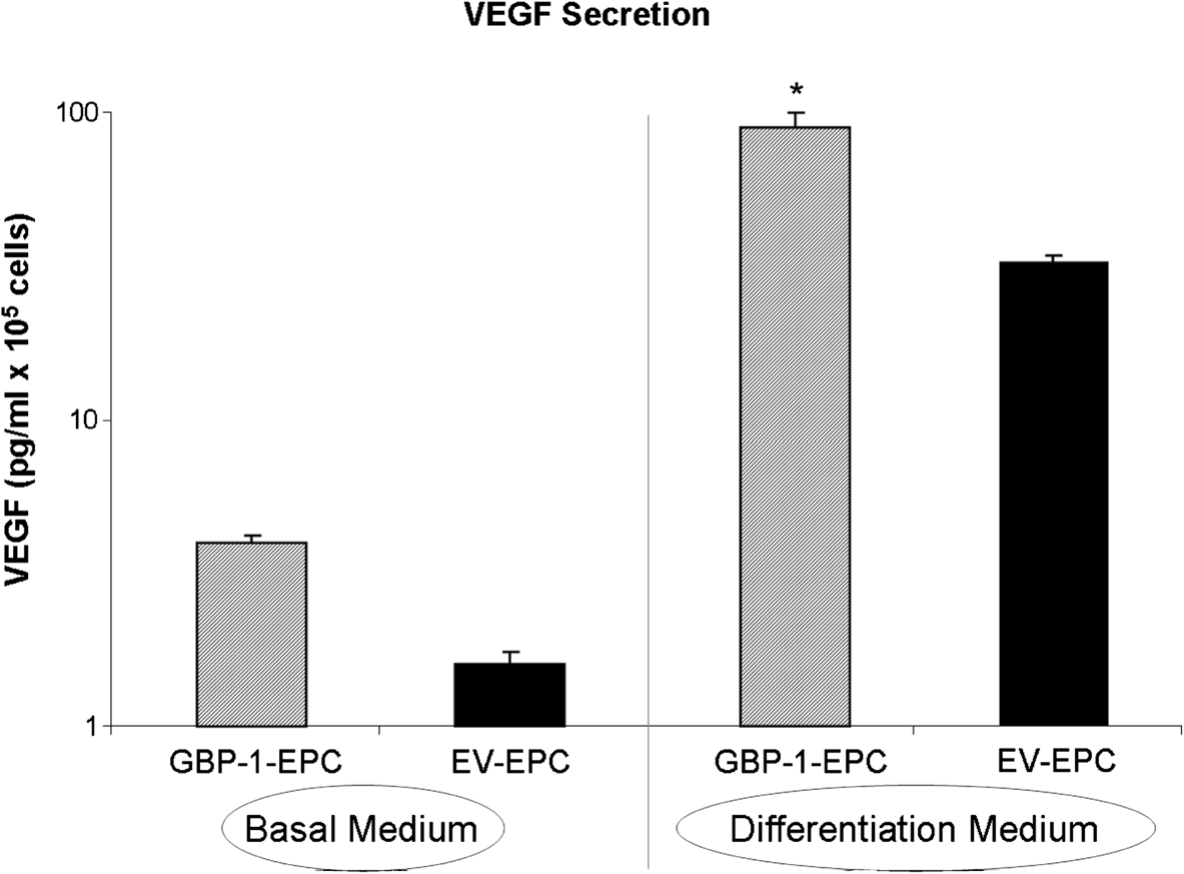
**VEGF secretion from cultured EPC in vitro incubated with basal medium (BM) or differentiation medium (DM). **Results are presented on a logarithmic scale. There is a significant increase in VEGF production upon differentiation in both EV-EPC and GBP-1-EPC. There is no significant difference between EV-EPC and GBP-1-EPC when basal medium is used. After incubation with differentiation medium, however, GBP-1-EPC secrete significantly higher levels of VEGF into the supernatant medium compared to EV-EPC. (p<0.05).

We quantified the ability of EV EPC and GBP-1 EPC to secrete VEGF under incubation with basal medium as well as differentiation medium. No significant difference was noticed when basal medium was used (1.25 ± 0.01 pg/ml/10^5^ cells vs. 1.33 ± 0.01 pg/ml/10^5^ cells, respectively, Figure 1). However, differentiation medium induced a significant stimulation of VEGF secretion in EV EPC and even more so in GBP-1 EPC (p<0.05). VEGF secretion from GBP-1 EPC was 86 ± 2 pg/ml/10^5^ cells, while secretion from EV EPC was significantly lower, reaching 39 ± 3 pg/ml/10^5^ cells (p<0.05, Figure 1).

### Surgery and animals

All animals tolerated the surgical procedure well without any major complications such as thrombosis of the vascular axis, extrusion of the chambers, infections or hematomas. Patency of the AVLs was assessed during the explantation after Microfil perfusion and during histological examination, revealing no signs of thrombosis.

### Macroscopic appearance

After explantation, vascularization of matrices was visible by yellow appearing filling of vessels with Microfil solution.

Macroscopic inspection revealed relatively stable fibrin clots at day 14. There was a slight macroscopic difference between the groups, with the AV loops containing EV EPC showing slightly more signs of clot degradation compared to EPC GBP-1 clots. There was some vascularization of matrices detectable. All AV loops were macroscopically patent upon explantation (Data not shown).

### Micro-CT

**Figure 2:**
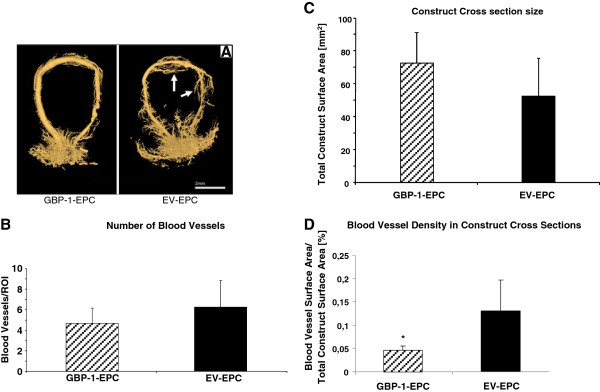
**A. Micro CT scans of explanted constructs. **At 14 days, micro-computed tomography demonstrated newly formed vessels, radiating from the arteriovenous loop in both groups. There appeared to be less neovessel formation in the GBP-1-EPC group (left) compared to EV-EPC (right). One particular source of neovessel formation appears to be the interposed graft from the contralateral femoral vein (arrow). **Scale Bar = 3 mm. B.** Morphometric Analysis of Number of Blood Vessels. At the time of explantation after 2 weeks, there was no significant difference in the average number of blood vessels per region of interest. **N= 4 animals per group. 2 independent cross section analyses per animal and construct were performed, i.e. 8 values per group were obtained and evaluated including statistical analysis. C. **Morphometric Analysis of Construct Cross Section Size. At the time of explantation 2 weeks after surgery, GBP-1-EPC containing constructs had a larger cross section compared to EV- containing EPC, however, this difference was not statistically significant. **N= 4 animals per group. 2 independent cross section analyses per animal and construct were performed, i.e. 8 values per group were obtained and evaluated including statistical analysis. D. **Morphometric Analysis of Blood Vessel Density in Construct Cross Sections. Presented as the ratio of blood vessel surface area over total construct surface area, a significant reduction of the percentage of vascularized area in the GBP-1-EPC group compared to control EV EPC is noted. * p<0.05 vs. EV-EPC. **N= 4 animals per group. 2 independent cross section analyses per animal and construct were performed, i.e. 8 values per group were obtained and evaluated including statistical analysis.**

The vascular network could be visualized by micro-CT of the specimens (n = 4 per group; Figure 2). In both groups, neovascularisation was evident arising from the main vascular axis. There was a difference which was particularly evident in the area of the contralateral interpositional vein graft, where new blood vessels appeared more numerous and displayed more advanced growth away from the AV loop in the EV EPC group (Figure 2A). In contrast, GBP1 expression reduced vessel ingrowth away from the AV loop (Figure 2A).

### Histology

Morphometric analysis was based on histological cross sections of explanted constructs. We previously demonstrated that vessels perfused with Microlfil could be identified histologically by black-appearing filling of the lumina [9]. Moreover, the fibrovascular tissue evolving from the two central vessels, artery and vein, could be clearly detected and its margins were easily discernible from the adjacent fibrin matrix. No infections were noticed macroscopically and histologically as indicated by the absence of inflammatory cells. Apart from the quantitative differences in blood vessel number, construct surface area and blood vessel density there were no differences between the two study groups (Data not shown).

### Morphometric analysis

There was no significant difference in the numbers of blood vessels per region of interest (ROI) between the two groups (6.25 ± 2.56 blood vessels/ROI in EV EPC vs. 4.66 ± 1.46 blood vessels/ROI in GBP-1 EPC) (Figure 2B).

The total construct surface area measurement of cross sections from EV-EPC containing constructs was 52.25 ± 23.5 mm^2^, while there was an average of 73.25 ± 17.71 mm^2^ surface area for GBP-1 EPC. There was no statistically significant difference (p>0.05) (Figure 2C).

The ratio of total blood vessel surface area to total construct surface area as an indicator for blood vessel density within the construct revealed a significant reduction by more than three-fold of the percentage of vascularised area in the GBP-1-EPC group compared to control EV EPC (0.13 ± 0.06% vs 0.04 ± 0.01% vascularised total surface area per total construct surface area, n = 4 constructs per group; p<0.05, Figure 2D).

### Immunohistochemistry and apoptosis

**Figure 3:**
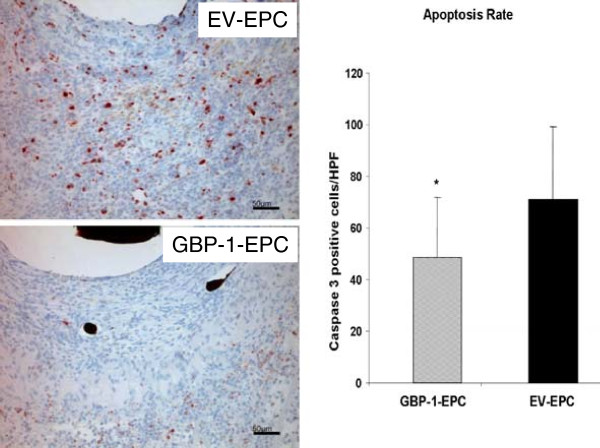
**Visualisation and Quantification of Apoptosis in Constructs by Caspase-3 IHC.** There was a significant reduction in the number of apoptotic cells seen in constructs containing GBP-1 EPC compared to EV-EPC. * P< 0.05 vs. EV EPC. **N= 4 animals per group. 2 independent cross section analyses per animal and construct were performed, i.e. 8 values per group were obtained and evaluated including statistical analysis. Scale Bar = 50 μm.**

Caspase-3 staining revealed a significant difference in apoptosis rate, with 71 ± 28 Caspase 3-positive cells/ROI in EV EPC constructs vs. 48 ± 23 Caspase-3-positive cells/ROI in GBP-1 EPC, indicating a significant reduction in cell apoptosis in the AV loop in the presence of GBP-1 expression from transplanted EPC (p<0.05, Figure 3). Caspase-3 positive cells can be identified by their red staining.

### Fluorescence microscopy

**Figure 4:**
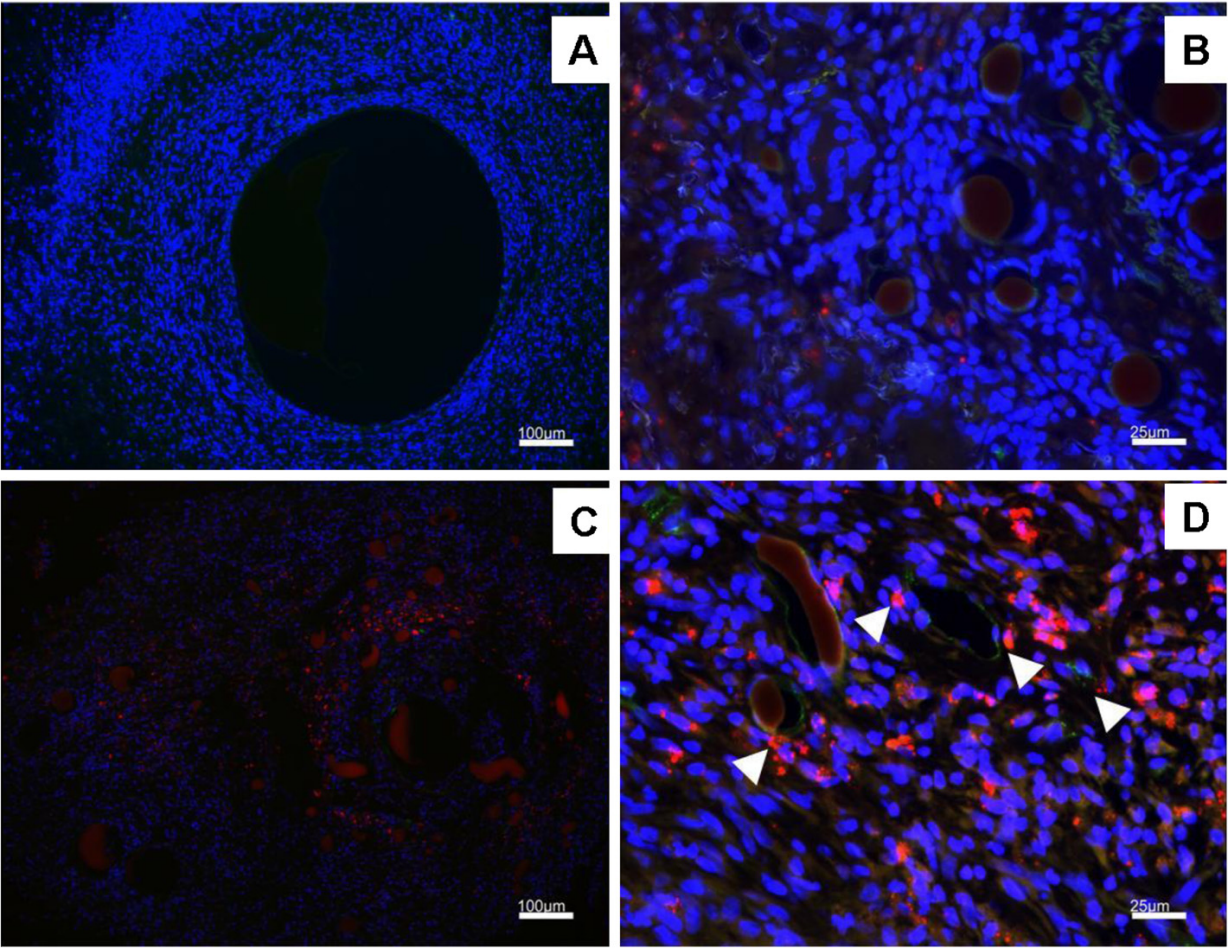
**Identification and Localisation of EPC. **Fluorescence staining for blood vessels in green with BS-1 lectin and transplanted DiI-labelled EPC in red. The number of GBP-1 EPC that can be found near the capillaries is much smaller (**A**) compared to numerous EV-EPC found in the near vicinity of newly formed blood vessels (**B**). **Scale Bar = 100 μm (A , C); Scale Bar = 25 μm (B, D).**

Fluorescence microscopy was carried out for constructs containing either GBP-1 EPC (Figure 4A, B) or EV EPC (Figure 4C, D). Two different magnifications show overview (Figure 4A, C or detail photograps (Figure 4B, D) of representative areas for both study groups. BS-1 lectin (green fluorescence) was used to label functional blood vessels. There was an increased number of DiI-expressing EV EPC (red fluorescence) in the close vicinity of small blood vessels (Figure 4D) while GBP-1 expressing EPC were not closely co-localized with blood vessels (Figure 4B). Equal distribution was shown for cells containing nuclei by DAPI staining (Data not shown).

## Discussion

The term “therapeutic vasculogenesis” was coined by Isner and Asahara [12] to imply that de novo blood vessel formation in adulthood can occur from differentiating endothelial progenitor cells derived from the bone marrow and peripheral blood. In the following years, tremendous efforts have been made in a multitude of experimental and clinical studies using EPC to stimulate blood vessel formation in ischemic conditions such as myocardial infarction and limb ischemia [13]. Even though a number of clinical trials have demonstrated encouraging results and therapeutic effects of EPC in patients with myocardial ischemia [14] and peripheral vascular disease [15], conflicting studies have raised questions regarding mechanisms of action and efficacy of EPC in different pathological settings [16]. Indeed, it has been shown that EPC numbers and function are decreased in conditions such as diabetes [17] and rheumatoid autoimmune disorders [18,19]. Serum levels of proinflammatory cytokines including TNF-α, IFN-γ, and IL-1α are increased in patients with rheumatoid arthritis and other autoimmune inflammatory disorders [19]. These cytokines exhibit an inhibitory effect on proliferation and migration of endothelial cells through the GTPase GBP-1 [5-8]. In this context, GBP-1 has been identified as a potent anti-angiogenic mediator in colorectal carcinoma, where a positive correlation was found between a high GBP-1 expression from tumor endothelial cells and reduced angiogenesis within the tumor [20].

 We recently investigated potential effects of GBP-1 on endothelial progenitor cell function [9] and aimed to identify antiangiogenic mechanisms that could explain EPC dysfunction in the inflammatory state [21] and associated cardiovascular disease [22]. The well characterized murine EPC line T17b was stably transfected with GBP-1. Compared to T17b expressing empty vector as a control (EV EPC), GBP-1 was sufficient to induce premature differentiation in EPC, while proliferation and migration were inhibited *in vitro*[9]. In the AVL model, GBP-1 EPC migration towards the central vessels was significantly reduced compared to EV EPC controls [9].

In the present study, we further characterized the influence of GBP-1 on EPC and construct morphometry in a developing three dimensional vascular network in the AVL model. Further functional *in vitro* testing revealed increased VEGF secretion from GBP-1 EPC compared to EV EPC under differentiation, while there was no difference in VEGF secretion under incubation with basal medium. Hence, VEGF secretion is associated with differentiation, which occurs also in vivo when EPC differentiate *in situ* within the construct. This is a further corroboration of our previous finding that premature differentiation *in vivo* prevented GBP1 EPC migration within the AVL construct towards the central blood vessels [9].

*In vivo,* Micro CT scans showed *neo*-vessel formation arising from the main vascular axis in both groups. This finding was confirmed by morphometric analysis of conventional histological cross sections. There was visible blood vessel ingrowth towards the center of the construct from the interposed contralateral venous graft in the EV EPC group which was not present in the GBP-1 EPC group. This may indicate that GBP-1 may not only be an intracellular inhibitor of angiogenesis in the endothelial cells itself, but prevent vascular ingrowth towards regions with high levels of GBP-1 expression. Even though we noticed increased VEGF expression in GBP-1-EPC after differentiation *in vitro* by ELISA, the presence of GBP-1 *in vivo* may locally interfere with the proangiogenic stimulus associated with the expression of VEGF in our in vivo model. It may be an unexpected finding that the transplanted GBP-1-EPC secrete substantial amounts of VEGF *in vitro,* yet fail to stimulate angiogenesis efficiently *in vivo*. We previously demonstrated, however [9], that concomitant GBP-1 exression inhibits EPC migration towards the site of active angiogenesis in our AVL microsurgical model. This may preclude EPC participation in neoangiogenesis since GBP-1 are unable to home to the region where they could exhibit an effect on the vascularisation process. Indeed, EPC may in this case secrete increased VEGF levels, unable to migrate to the site where they could exhibit a biological effect towards angiogenesis. On the other hand, we demonstrated that EPC lacking GBP-1 were often located in the close vicinity of newly formed blood vessels, supporting the assumption that they secrete proangiogenic growth factors *in vivo*, supporting neovascularisation through a paracrine fashion.

Our experimental results are supported by clinical findings in colorectal cancer patients where increased GBP-1 expression from endothelial cells was associated with a reduction in angiogenesis [20].

 We measured a 29% reduction in cross section size of AVL constructs containing EV EPC compared to GBP-1 EPC constructs. Our group previously demonstrated reduction of cross section size of the AVL constructs when recombinant growth factors VEGF and bFGF were suspended within the fibrin matrix, leading to increased angiogenesis and fibrovascular tissue formation [23]. These findings indicate that cross section size of the construct can be used as an indicator for shrinkage due to increased fibrovascular tissue inside formation. This supports the hypothesis that GBP-1 prevented blood vessel formation and hence concomitant degradation of the fibrin construct. Previous work from our group by Arkudas et al. [23] demonstrates that fibrin loaded with recombinant VEGF does effectively support blood vessel growth in a dose dependent manner. In this setting, however, VEGF is likely homogenously distributed within the fibrin gel, which does not appear top be the case with the transplanted EPC in our study. The presence of GBP-1 reduces EPC migration capacity, and areas where VEGF expression is particulary high do exert a negative chemotactic effect on GBP-1 expressing cells, i.e. GBP-1-EPC cannot locate to the vicinity of intense growth of new blood vessels. To further elucidate the impact of GBP-1 on the angiogenic stimulus in our AVL model, we assessed the ratio of total blood vessel surface area to total construct surface area in complete representative construct cross sections. Of note, we found a significant reduction in the percentage of blood vessel surface area in relation to the total construct, indicating a reduced vascular density attributable to GBP-1 expression from EPC. Unfortunately, it is difficult to pinpoint the exact mechanisms by which EPC exhibit their effects in this study. Based on the distribution pattern of fluorescently labelled EV-EPC, it appears that they exerted their proangiogenic effect mostly in a paracrine fashion rather than being directly integrated into newly formed blood vessels. Overexpression of GBP-1 in EPC has been shown to reduce their migratory capacity as well as their potential for capillary formation [5]. We also previously confirmed that GBP-1 inhibited vessel-directed migration of EPC at the tissue level using the rat arterio-venous loop model [9]. Hence, we stipulate that several mechanisms contribute to reduced blood vessel formation of GBP-1 EPC. On the one hand, it is cell-inherent due to the antiangiogenic effect of GBP-1. On the other hand, GBP-1 EPC appear to be less capable to localize to areas of intense angiogenesis where they are most needed to exert their function.

It is also known that GBP-1 has an anti-apoptotic effect on endothelial cells. The rate of apoptosis in our construct, as detected by Caspase-3 staining, was significantly reduced in the GBP-1 EPC - Group. This may indicate an antiapoptotic effect induced by GBP-1. This hypothesis is supported by findings from Pammer et al. where human umbilical vein endothelial cells (HUVEC) were protected from apoptosis upon transfection with an expression vector for GBP-1 [24]. They further attributed the antiangiogenic effect to the fact that GBP-1 induces senescence in endothelial cells rather than apoptosis.

## Conclusion

Taken together, this is the first study demonstrating the potential of the antiangiogenic proinflammatory large GTPase GBP-1 to alter EPC behaviour, reduce apoptosis rate and blood vessel density after transplantation into a developing vascular network. These results may open a new option towards antiangiogenic treatment strategies to inhibit undesired angiogenesis and target inhibition to defined regions of angiogenetic activity in cancer and other disease conditions.

## Methods

### Cell culture and stable transfection

The endothelial progenitor cell line T17b has been previously isolated from mouse embryos and was cultured as previously described [25]. Briefly, cells were maintained in DMEM Glutamax (Invitrogen, Carlsbad, CA, USA) containing 20% fetal bovine serum, 100 U/ml Penicillin, 100 μg/ml Streptomycin, 0.1mM β-Mercaptoethanol, 1 mM non-essential amino acids and 2 mM HEPES buffer pH 7.5 (all purchased from Invitrogen). As opposed to this basal medium (BM), cell differentiation was induced by incubation with differentiation medium (DM) where BM was supplemented with 0.5 mM dibutyryl cyclic AMP (cAMP) (Sigma Aldrich Chemie, Schnelldorf, Germany) and 1 μM retinoic acid (RA) (Sigma Aldrich Chemie) for 3 consecutive days. Cells were maintained in T-75 culture flasks (Nunc, Wiesbaden, Germany) coated for 2 hours with 0.1% bovine skin gelatine, type B (Sigma Aldrich, Taufkirchen, Germany) in phosphate-buffered saline (PBS) (Biochrom AG, Berlin, Germany). The cells were subcultured using 1 x 0.5 g/L trypsin and 0.2 g/L ethylene-diamine-tetra-acetic acid in HBSS (trypsin/EDTA) (PAA, Pasching, Austria) and passaged in a 1:8 ratio. Cells were counted using the CASY cell counting system (Schärfe System, Model DT, Reutlingen, Germany). Cells were tested for mycoplasma infection on a regular basis using MycoAlert (Cambrex, Charles City, USA). All results were negative.

EPC expressing the transgene GBP-1 were generated through clonal selection as previously described [9]**.** Briefly, 1 x 10^5 ^cells per well were seeded in precoated 6-well plates. After 24 hours, transfection was carried out using the Effectene transfection reagent (Quiagen, Hilden, Germany) with 2 μg Plasmid DNA. pMCV-2.2 and pMCV-2.2-Flag-GBP-1 plasmids. A G418 resistance gene was employed in both plasmids and selection was carried out for 10 days with 500 μg/ml G418 (PAA) followed by successful selection of an EPC clone expressing GBP-1 stably and at biologically siginifcant levels [9].

To quantify VEGF secretion from EPC expressing GBP-1 and control EPC, cells were seeded at 5 x 10^5 ^cells and incubated with either differentiation medium or basal medium for 4 subsequent days where medium was changed on a daily basis. In either case, T17b EPC culture supernatant was collected at day 4 24 hrs after the last medium change and VEGF secretion was quantified using a VEGF ELISA kit (R&D Systems, Minneapolis, MN, USA) according to the manufacturer’s instructions. Experiments were carried out in triplicates and repeated twice.

### Labeling of EPC

EPC were labelled with the fluorescent carbocyanine DiI dye (Molecular Probes) prior to transplantation as previously described [15] in order to facilitate tracking and localisation. In brief, before cellular transplantation, EPCs in suspension were washed with PBS and incubated with DiI at a concentration of 2.5 μg/ml PBS for 5 minutes at 37°C and 15 minutes at 4°C. After two washing steps in PBS, the cells were resuspended in DMEM Glutamax medium.

### Experimental design *in vivo*

Two experimental groups employing 4 animals per group were investigated. Both groups received EPC suspended in the fibrin matrix where the AVL was embedded. One group of animals received GBP-1-EPC, the other group received EV-EPC as a control. At day 14 after surgery experiments were terminated and constructs explanted for further analysis.

### Animals

Male Lewis rats (Charles River Laboratories, Sulzfeld, Germany) were used and German regulations for the care and use of laboratory animals were observed throughout the entire study.

All experiments were approved by the animal care committee of the University of Erlangen and the Government of Mittelfranken, Germany (Approved Protocol 54–2532.1-28/09).

Animals were housed in the veterinary care facility of the University of Erlangen Medical Center and submitted to a 12-hour dark–light cycle with free access to rodent standard diet (Altromin, Hamburg, Germany) and tap water.

### Surgical procedures

The animals were anesthetized by general anesthesia with Isoflurane (Baxter, Unterschleißheim, Germany). The AVL was created as described previously [26]. Briefly, through longitudinal skin incisions both femoral vessels were exposed and dissected from the groin to the knee. A 20 mm vein graft was harvested from the right femoral vein. Interposing this graft in retrograde fashion an arteriovenous loop was created between the left femoral artery and vein by end-to-end anastomosis using 11–0 sutures (Ethicon, Norderstedt, Germany). Chambers containing 5x10^6^ EV-EPC or GBP-1-EPC suspended in 500 μl of a clinically approved fibrin gel (Tissucol, Baxter, Unterschleißheim, Germany) with a fibrinogen concentration of 10 mg/ml, a thrombin concentration of 2 IU/ml and an aprotinin concentration of 1500 KIE/ml to delay fibrinolysis were implanted. In detail, the chamber was filled with 250 μl fibrin gel – EPC suspension, and the AV loop was passed around the four plastic sticks. Then, the chamber was filled with the second half of the fibrin-EPC suspension to a total volume of 500 μl, the lid was closed, and the chamber was fixed in the groin using Prolene 3–0 (Ethicon, Norderstedt, Germany) sutures. For analgesia buprenorphin (0.3 mg/kg rat weight, Temgesic, Essex Chemie AG, Luzern, Switzerland) was administered postoperatively. Also all animals received 0.2 ml benzylpenicillin-benzathin (Tardomycel, Bayer, Leverkusen, Germany), and heparine (80 I.U./kg Liquemin, Ratiopharm, Germany). 30 minutes before animals were sacrificed, they received an intravenous injection of of 500 μg Bandeirea Simplicifolia Lectin (BS-1 l) conjugated with FITC (Vector Laboratories). 4 constructs per group were examined.

### Explantation procedure and microfil® injection

At 2 weeks after the initial AV loop implantation rats were anesthetized by general anesthesia with Isoflurane and the aorta and the inferior vena cava were exposed through a median incision from the xiphoid process to the pubic symphysis. After cannulation of the aorta the vena cava was cut and the vascular system of the rat was flushed with 200 ml heparinized Ringer solution (100 I.U./ml). The caudal vascular system was then rinsed with 20 ml yellow Microfil® (MV-122) containing 5% of MV Curing Agent (both from Flowtech, Mass., USA). Finally the rats were cooled at 4°C for 24h and the constructs were harvested in toto and fixed in 3.5% formalin solution.

### Micro-computed tomography (micro-CT)

One specimen per group was studied using mirco-CT for qualitative evaluation. Therefore the tube containing the explanted construct was placed on the table of a Micro-CT scanner (FORBILD, High Resolution Micro-CT, Erlangen, Germany). Scanning parameters were as follows: tube voltage 40 kV, 250 μA, 15 μm voxel size, tube-detector-distance: 250 mm.

### Histological and statistical analysis

4 constructs per group were explanted *in toto* with the surrounding tissue. After fixation in 3.5% formalin solution, constructs were dehydrated in graded ethanol and embedded in paraffin. 3 μm cross sections were obtained using a Leica microtome (Leica Microsystems, Bensheim, Germany) where two standardized planes were defined (1 mm proximal and 1 mm distal of the central plane) rectangular to the longitudinal axis of the AV loop.

Hematoxylin eosin (HE) staining was performed according to standard protocols. Immuno-histochemical staining was performed using the fluorescent Bandeiraea Simplicifolia agglutinin (BS-1) and Lectin. Fluorescence microscopy was carried out to detect EPC labelled with Di-I *in vitro* prior to implantation and ingrowing blood vessels decorated with BS-1 lectin which was intravenously injected immediately prior to sacrification of the animals.

The images were evaluated by an independent and blinded observer. All images of H&E stained cross sections were generated with a Leica microscope and digital camera using 25 x magnification.

### Immunohistochemistry

Lectin: The lectin Bandeiraea Simplicifolia agglutinin (BS-1) was used for immunohistochemical detection of rat endothelial cells [4]. Paraffinated sections were submitted to a xylol/ethanol sequence, rinsed in PBS followed by blocking of endogenous peroxidise activity in H_2_O_2_ for 10 minutes. Blocking was completed by incubation with avidin and biotin 15 minutes each (Vector Laboratories, California) as well as 5% goat serum for 1 hour. Overnight incubation was performed with Biotinylated lectin (BS-1, Sigma) in a 1:100 dilution in PBS overnight at 4°C. Slides were rinsed in PBS between incubation steps. Detection was achieved by incubation with Streptavidin AB Complex/HRP (Dako GmbH, Germany) for 30 minutes followed by development with DAB + Chromogen (Dako GmbH). Cardiac muscle sections were used as positive controls, while absence of lectin served as negative control.

Caspase-3: Cleaved Caspase-3 staining was used for immunohistochemical detection of apoptosis. After deparaffination antigen retrieval was performed by boiling of sections in TRS-buffer pH 6 (Dako GmbH) for 2,5 minutes using a pressure cooker und followed by blocking of endogenous peroxidase activity in H2O2 for 10 minutes. Blocking was completed by incubation with avidin and biotin 20 minutes each (Vector Laboratories, California) as well as 1% bovine serum albumin (BSA) for 1 hour at room temperature. Slides were rinsed in PBS between incubation steps. Detection was achieved by incubation with primary polyclonal rabbit antibody anti-cleaved Caspase-3 (DCS-diagnostics, Hamburg, Germany) at a dilution of 1:100 for 40 min at 37°C. A secondary goat anti rabbit antibody (Vector laboratories, Burlingame, USA) at a dilution of 1:200 was subsequently used for 30 min incubation at room termperature. After washing with TRIS buffer, ABC solution (Vector Laboratories, Eching, Germany) was used applied for 30 min followed by Tyarmid enhancement using the NEN kit (Perkin Elmer, Rodgau, Germany) for 15 min at 1:50 dilution. Thereafter, ABC solution (Vector laboratories) was applied again for 15 min. Followed by AEC for 2 min and Hemalaun staining.

### Morphometry

Morphometric analysis was performed by blinded observers. In brief, total cross section surface area of the construct was quantified for both groups, GBP-1 EPC and control EPC, according to the method previously described by Arkudas et al. [27]**.** In brief, three-mm cross sections were obtained from 2 standardized planes. Both planes were oriented rectangular to the longitudinal axis of the AV loop. One plain was located 1 mm proximal, the other 1 mm distal to the transverse midline. Hence, 2 cross section analyses per animal and construct were performed, resulting in a total of 8 cross sections of the entire construct per each of the two experimental groups. The mean value was calculated for each group and each parameter. To determine the number of blood vessels per cross section, blood vessel distribution, and total blood vessel surface area all H&E stained cross sections were split into 14 sectors, 6 at the periphery, 4 at the central part of the section and each 2 at the artery and vein [27]. Afterwards, the images of each sector (200x magnification) were rendered bimodal using a standardized threshold (WinQ, Leica Microsystems, Bensheim, Germany) followed by the calculation of the above mentioned parameters.

### Fluorescence microscopy

Images were generated with a Leica microscope using 200 x magnification. Appropriate filter systems for detection of the double fluorescence labelling was used (red – Di-I; green – BS-1 lectin).

### Statistical analysis

Results are presented as mean ± standard deviation. Statistical analysis was carried out GraphPad Prism software (GraphPad Software, San Diego, USA). Two-tailed unpaired student’s t-test was applied for statistical analysis. A p value <0.05 was determined statistically significant.

## Competing interests

There were no competing interests for any of the authors of this manuscript.

## Authors’ contributions

OB, MH, EN and UK designed Research. OB, MH and KA performed research. OB, MH, AA, CT, JPB, EN and MS analyzed data. OB and UK wrote the manuscript. All authors read and approved the final manuscript.
